# Application of a Novel Solid Silver Microelectrode Array for Anodic Stripping Voltammetric Determination of Thallium(I)

**DOI:** 10.3390/molecules30214220

**Published:** 2025-10-29

**Authors:** Mieczyslaw Korolczuk, Mateusz Ochab, Iwona Gęca

**Affiliations:** Institute of Chemical Sciences, Faculty of Chemistry, Maria Curie Sklodowska University, 20-031 Lublin, Poland; mieczyslaw.korolczuk@mail.umcs.pl (M.K.); mateusz.ochab@mail.umcs.pl (M.O.)

**Keywords:** silver microelectrode array, thallium, anodic stripping voltammetry, determination, environmental monitoring

## Abstract

The article reports for the first time the application of a solid silver microelectrode array for the anodic stripping voltammetric determination of thallium(I) ions (Tl(I)). The microelectrode properties of the presented sensor were tested. The proposed solid metal microelectrode array is characterized by its eco-friendly nature due to the use of non-toxic electrode material. The advantage of this procedure is that no surface modification of the microelectrode was required. The optimization of the procedure for determining Tl(I) was performed. The experimental parameters (e.g., pH of supporting electrolyte, conditions of activation step, potential and time of deposition, effects of possible interferences) were investigated. The dependence of the thallium peak current on its concentration was linear in the range from 5 × 10^−10^ to 1 × 10^−7^ mol·L^−1^ (deposition time of 120 s). The estimated detection limit was 1.35 × 10^−10^ mol·L^−1^. The repeatability of the procedure expressed as RSD% for a Tl(I) concentration of 2 × 10^−8^ mol·L^−1^ was 3.6% (n = 5). The proposed procedure was applied for determining Tl(I) in certified reference materials and for studying recovery in the environmental water sample. The obtained results indicated the possibility of an analytical application of the elaborated procedure in practice.

## 1. Introduction

The determination of thallium is of great importance due to the metal’s high toxicity and potential possibility of its bioaccumulation [1,2], e.g., in environmental and biological samples. Thallium levels monitoring may help in assessing thallium contamination, exposure levels and potential health risks.

To date, thallium has been reported to be quantified by various analytical methods, e.g., flame atomic absorption spectrometry [3,4], graphite furnace atomic absorption spectrometry [5], inductively coupled plasma mass spectrometry [6,7], high-performance liquid chromatography with inductively coupled plasma mass spectrometry [8,9] and X-ray fluorescence spectrometry [10,11].

For the determination of thallium, stripping voltammetry is often the method of choice and is competitive with the methods mentioned above due to its portable and relatively inexpensive instrumentation, short duration of measurements and the possibility of providing speciation analysis. To date, thallium has been determined by stripping voltammetry through the use of several different working electrodes. The following electrodes can be noted: hanging mercury drop electrode [12], mercury film electrode [13], bismuth film electrode [14,15], bismuth bulk annular band electrode [16], mechanically renewed Bi-graphite electrode [17], solid bismuth microelectrode [18], antimony film electrode [19], graphite microelectrode [20], modified carbon paste electrodes [21,22], screen-printed electrodes [23,24], silver single crystal electrodes [25], silver-gold alloy electrode [26,27], metal modified solid gold microelectrode array [28], glassy carbon electrode modified with silver nanostructures reduced and stabilized with dextrin [29] and thiol functionalized multiwall carbon nanotubes [30]. However, in some cases, the application of some of the aforementioned electrodes is limited due to the high toxicity of electrode materials [12,19], while in other cases, this is due to the complicated, long or time-consuming electrode preparation stage [30]. As illustrated in Table 1, a comparison is made between the analytical parameters of the voltammetric procedure presented in the article and those of previously reported procedures that employed a variety of working electrodes.

In the present paper, the solid silver microelectrode array is reported for the first time for analytical purposes. The proposed microelectrode array was applied for the anodic stripping voltammetric determination of Tl(I) ions. The presented microelectrode array is an environmentally friendly sensor because of the use of a non-toxic metal for its fabrication. Moreover, in the proposed procedure, no modification of the electrode surface is required (e.g., metal pre-plating), which makes the procedure ecological, simple and relatively short. The conditions for the determination of Tl(I) ions at the solid silver microelectrode array were optimized. The presented procedure was applied for the analysis of certified reference materials (TM 25.5 and TM 26.5) and for studying recovery in the natural water sample. The agreement between the obtained results and certified values, as well as satisfactory recovery values, confirmed the correctness of the analytical procedure.

## 2. Results

**Figure 1:**
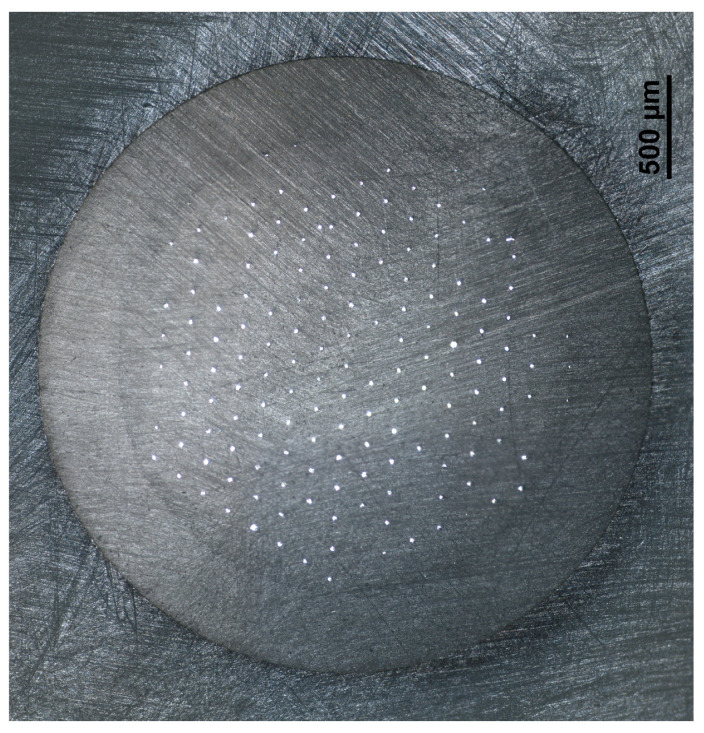
A real image of the surface of a solid silver microelectrode array.

**Figure 2:**
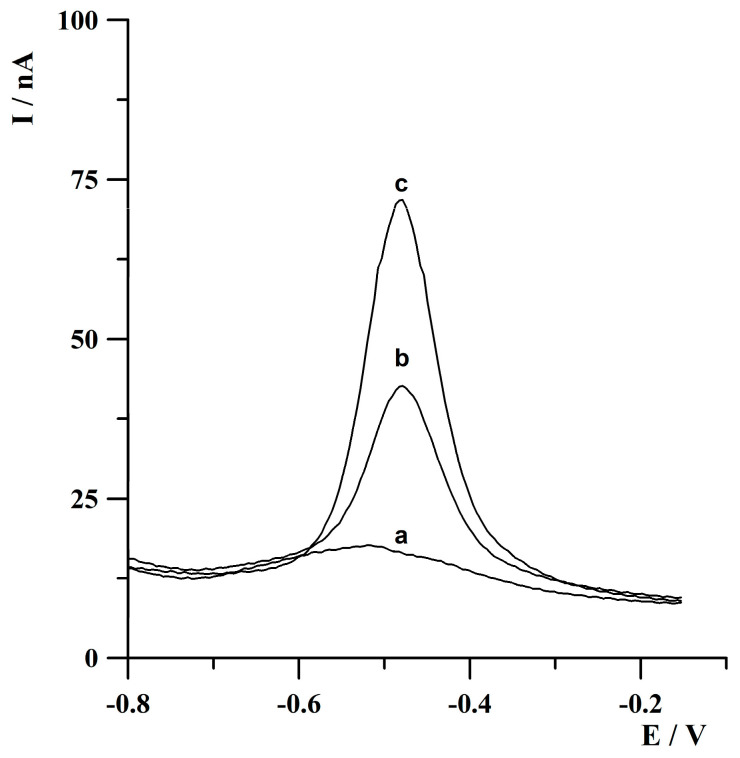
Anodic stripping voltammograms obtained for (a) blank; (b) 5 × 10^−8^ mol·L^−1^ Tl(I) without solution stirring during deposition step; (c) 5 × 10^−8^ mol·L^−1^ Tl(I) with solution stirring during deposition step. Activation conditions: −3.0 V, 2 s. Deposition conditions: −0.9 V, 60 s. The measurements were conducted from an acetate buffer solution of a pH of 4.2. The solution was deoxygenated for 5 min before the measurements.

In this article, a novel solid silver microelectrode array and its exemplary application in the determination of Tl(I) is presented for the first time. The real view of the surface of the solid silver microelectrode array, taken by an inverted microscope MA200 Inverted Metallographic Microscope Nikon (Tokyo, Japan), is shown in Figure 1. According to the literature data [31], one of the ways of studying the microelectrode properties of a tested electrode of small dimensions is a comparison of the obtained analytical signals recorded after the deposition/accumulation step carried out in mixed and unmixed solutions. The observed two- to three-fold lower heights of the recorded peaks under quiescent conditions indicate effective spherical diffusion occurring at the surface of the tested electrode and, consequently, on the microelectrodes’ properties. These observations are in contrast with the results obtained using working electrodes of conventional sizes, where the observed differences in the values of analytical signal obtained from stirred and unstirred solutions are much more significant. The possibility of conducting the measurements without stirring the solutions during the deposition/accumulation step with satisfactory analytical responses can potentially be used for the analysis of very small volume samples, as well as for analysis under field conditions. The obtained results of the experiment concerning the testing of microelectrode properties of the presented array, shown in Figure 2, confirmed the microelectrode characteristics of the proposed sensor.

### 2.1. The Effect of a Solution Deoxygenation

**Figure 3:**
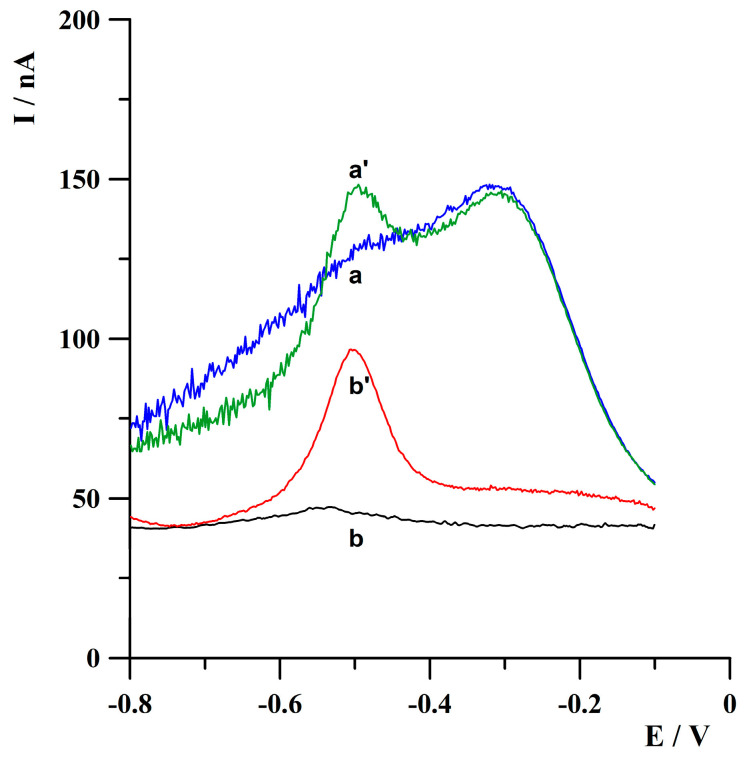
Anodic stripping voltammograms obtained for blank (a,b) and 5 × 10^−^^8^ mol·L^−^^1^ Tl(I) (a’,b’). The measurements were carried out without solution deoxygenation (a,a’) and after 5 min solution deoxygenation (b,b’). Activation conditions: −3.0 V, 2 s. Deposition conditions: −0.9 V, 60 s. The measurements were conducted from an acetate buffer solution of a pH of 4.2.

During preliminary studies, it was observed that deoxygenation of the analyzed solution allowed us to obtain well-shaped, easy to measure and interpret thallium analytical signals, and also lowered the background level. The second step of oxygen reduction at a potential of thallium oxidation can explain this observation. The results of preliminary experiments conducted from a solution after or without its deoxygenation are presented in Figure 3. Unless otherwise stated, further measurements were performed from deoxygenated solutions.

### 2.2. The Effect of pH and Concentration of Acetate Buffer and Na_2_EDTA

#### 2.2.1. pH Optimization Study

**Figure 4:**
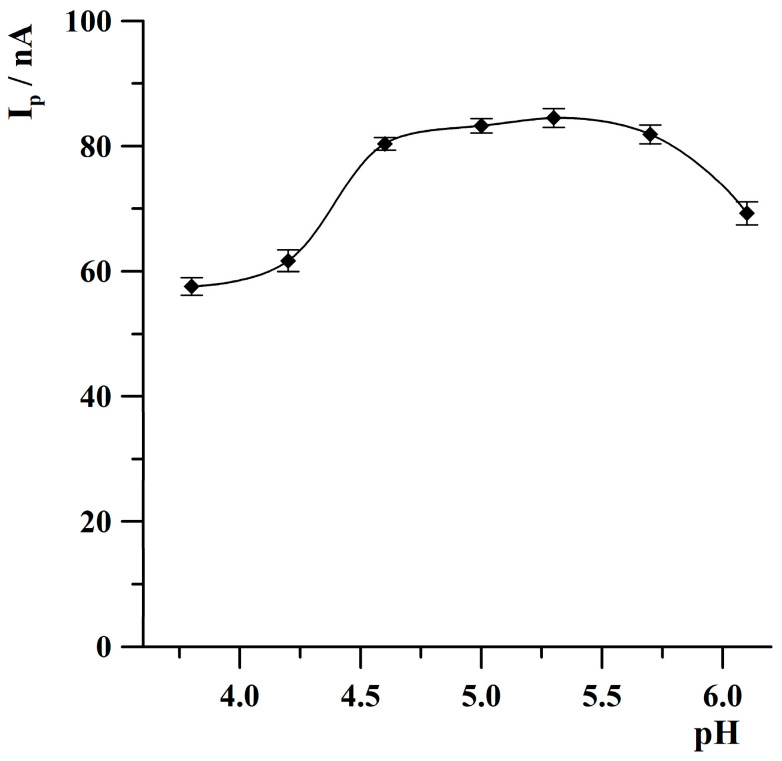
The effect of the pH of the acetate buffer on the thallium peak height. Concentration of Tl(I): 2 × 10^−^^8^ mol·L^−^^1^. Activation conditions: −3.0 V, 2 s. Deposition conditions: −0.9 V, 120 s. The error bars represent the standard deviation (n = 3).

The acetate buffer was used for the research as a main component of the supporting electrolyte. The study for selecting the appropriate value of the pH of the acetate buffer was performed from a solution containing Tl(I) at a concentration of 5 × 10^−8^ mol·L^−1^. The pH value was changed from 3.8 to 6.1 by an appropriate addition of 2 mol·L^−1^ NaOH. The results obtained during this study are illustrated in Figure 4. It was observed that the thallium peak current increased from a pH of 3.8 to 4.6, attained an almost constant value within the range from 4.6 to 5.7, and then slightly decreased at a pH of 6.1. Based on the results presented in Figure 4, it can be concluded that the most favourable value of pH was in the range from 5.3 to 5.7, and further research was conducted at a pH of 5.3. Anodic stripping voltammograms obtained during the study of pH optimization of the procedure of Tl(I) determination are illustrated in Figure S1 in the Supplementary Materials.

#### 2.2.2. Optimization of the Concentration of an Acetate Buffer

The influence of a concentration of an acetate buffer of a pH of 5.3 on the thallium peak current was studied within the range from 0.01 to 0.2 mol·L^−1^. The concentration of Tl(I) was 2 × 10^−8^ mol·L^−1^. The results obtained during this study are illustrated in Figure S2 in the Supplementary Materials. It was observed that the thallium peak current increased from 0.01 to 0.05 mol·L^−1^, attained the highest value at a concentration of 0.05 mol·L^−1^, and slightly decreased at higher concentrations of an acetate buffer. Further studies were carried out at the acetate buffer concentration of 0.05 mol·L^−1^.

#### 2.2.3. Optimization of the Concentration of Na_2_EDTA

The influence of a concentration of Na_2_EDTA was tested in the range from 0 to 10 mmol·L^−1^. The concentration of Tl(I) was 2 × 10^−8^ mol·L^−1^. The results obtained during this study are shown in Figure S3 in the Supplementary Materials. It was observed that the thallium peak current attained the highest value in the absence of Na_2_EDTA, and then slightly decreased with the increasing concentrations of the complexing agent. However, since Na_2_EDTA is a crucial component when determining Tl(I) in the presence of multivalent ions because of the masking of potentially interfering ions, the concentration of 2 mmol·L^−1^ was chosen for further studies. The rationale for incorporating EDTA in the determination of thallium ions in the presence of multivalent ions is illustrated in Figure S4 in the Supplementary Materials, employing the example of determining the analyte in the presence of a 100-fold excess of Pb(II). Our experimental observations can be explained by the fact that Na_2_EDTA forms with Pb(II) complexes, which are electrochemically inactive at the applied deposition potential. As a result, monovalent thallium ions may be determined without a significant interference effect caused by the potential presence of an excess of multivalent ions in the analyzed samples.

### 2.3. Optimization of Activation Conditions

**Figure 5:**
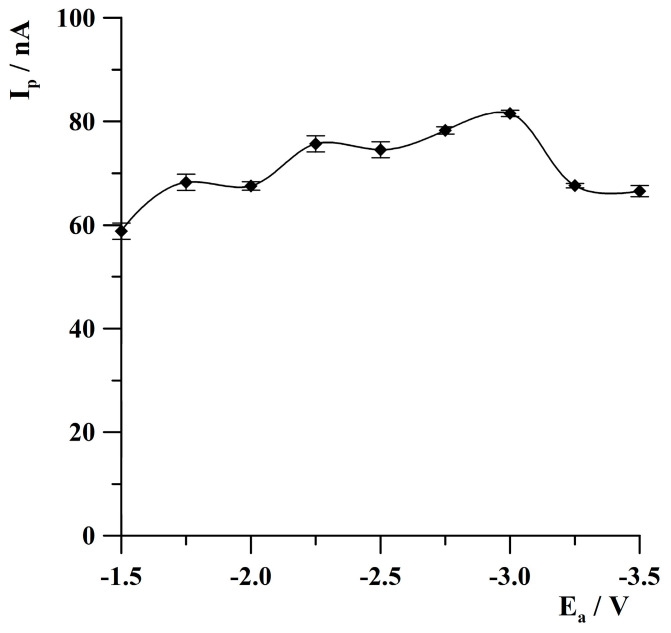
The effect of activation potential on thallium peak height. Concentration of Tl(I): 2 × 10^−8^ mol·L^−1^. Activation time: 2 s. Deposition conditions: −0.9 V, 120 s. The error bars represent the standard deviation (n = 3).

As it was previously stated [18,32], the activation step introduced to the standard measurement procedure is an important step when using solid metal electrodes because it improves both the quality of the recorded signals and the sensitivity of determinations. In the case of solid metal electrodes, this stage involves applying a short potential pulse lasting about several seconds with a highly negative potential value. Between the measurements, the electrode is exposed to air and may be partially oxidized. To eliminate the presence of oxides on the surface of the electrode, a short stage at a high negative potential is introduced, during which any oxides that can be formed are reduced to a metallic state. In the course of the presented studies, the effect of activation potential on thallium peak height was investigated in the range from −1.5 V to −3.5 V. The concentration of Tl(I) was 2 × 10^−8^ mol·L^−1^. The results are presented in Figure 5. As can be noted, the thallium analytical signal increased narrowly from −1.5 to −3.0 V, attained the highest value at −3.0 V, and then decreased at more negative activation potential. Further research was conducted at the activation potential of −3.0 V.

**Figure 6:**
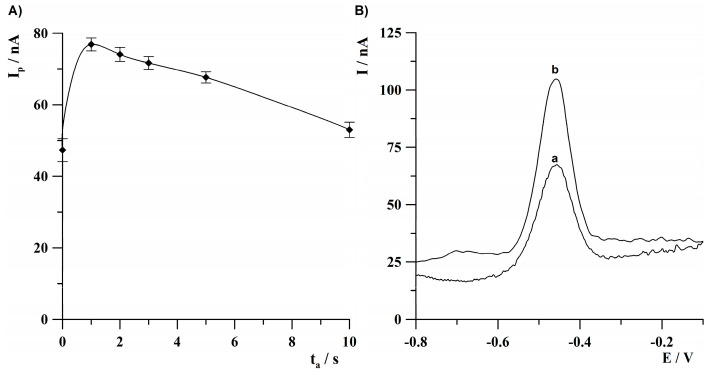
(**A**) The effect of activation time on thallium peak height. (**B**) Anodic stripping voltammograms obtained: (a) without applying an activation step (t_a_ = 0 s); (b) using a 1 s activation step. Concentration of Tl(I): 2 × 10^−8^ mol·L^−1^. Activation potential: −3.0 V. Deposition conditions: −0.9 V, 120 s. The error bars represent the standard deviation (n = 3).

The effect of activation time on the thallium analytical signal was investigated in the range from 0 to 10 s. The concentration of Tl(I) was 2 × 10^−8^ mol·L^−1^. The results are presented in Figure 6A. As can be seen, the thallium analytical signal obtained the highest value at 1 s of activation time and then decreased at longer activation times. Such a phenomenon can be explained by the reduction of hydrogen, which can partially block the working electrode’s surface. Based on the obtained results, for further studies, the activation time of 1 s was chosen as an optimal value. The benefit of using the activation step in the standard measurement procedure is presented in Figure 6B. As can be seen, the application of a short 1 s potential pulse of −3.0 V led to about a twofold amplification of the thallium analytical signal.

### 2.4. Optimization of Deposition Conditions

**Figure 7:**
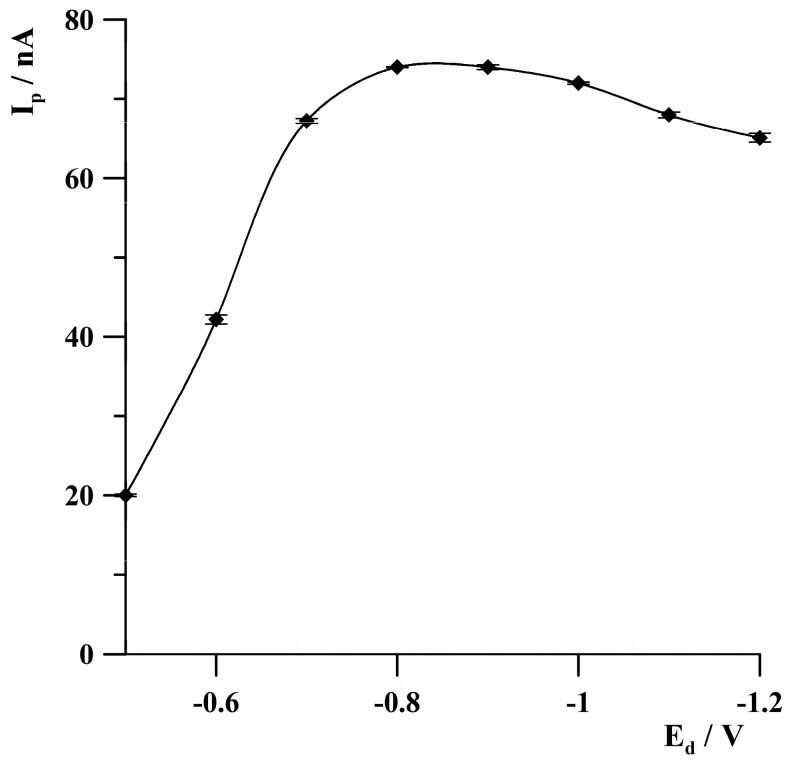
The effect of deposition potential on thallium peak height. Concentration of Tl(I): 2 × 10^−8^ mol·L^−1^. Activation conditions: −3.0 V, 1 s. Deposition time: 120 s. The error bars represent the standard deviation (n = 3).

The impact of deposition potential on the thallium peak height was studied in the range from −0.5 V to −1.2 V. The concentration of Tl(I) was 2 × 10^−8^ mol·L^−1^. The results are shown in Figure 7. As can be noted, the thallium signal increased from −0.5 to −0.8 V, attained the highest value in the range from −0.8 to −0.9 V, and then decreased narrowly at more negative deposition potential values. In order to avoid the deposition of potentially interfering metals that could potentially be observed at more negative potential values, further studies were performed at a deposition potential of −0.8 V.

**Figure 8:**
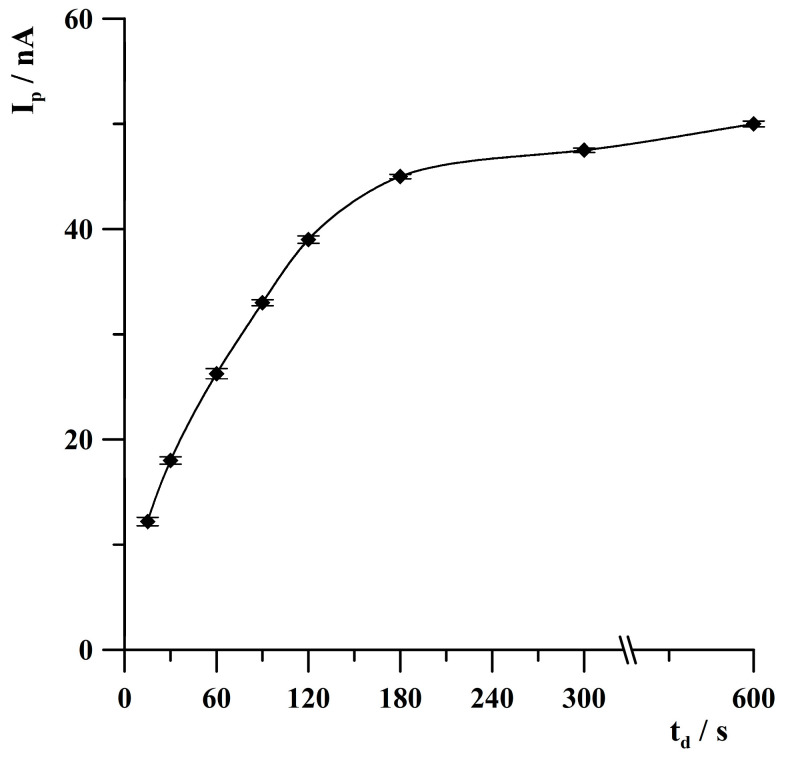
The effect of deposition time on thallium peak height. Concentration of Tl(I): 1 × 10^−8^ mol·L^−1^. Activation conditions: −3.0 V, 1 s. Deposition potential: −0.8 V. The error bars represent the standard deviation (n = 3).

The influence of deposition time on the thallium analytical signal was checked in the range from 15 to 600 s. The concentration of Tl(I) was 1 × 10^−8^ mol·L^−1^. The obtained results are presented in Figure 8. As can be seen, the thallium analytical signal increased almost linearly from 15 to 180 s and then increased much more slowly at longer deposition times. Based on the obtained results, further studies were conducted at a deposition time of 120 s; however, for obtaining higher sensitivity of determinations, longer deposition time is recommended.

### 2.5. Optimization of Frequency

**Figure 9:**
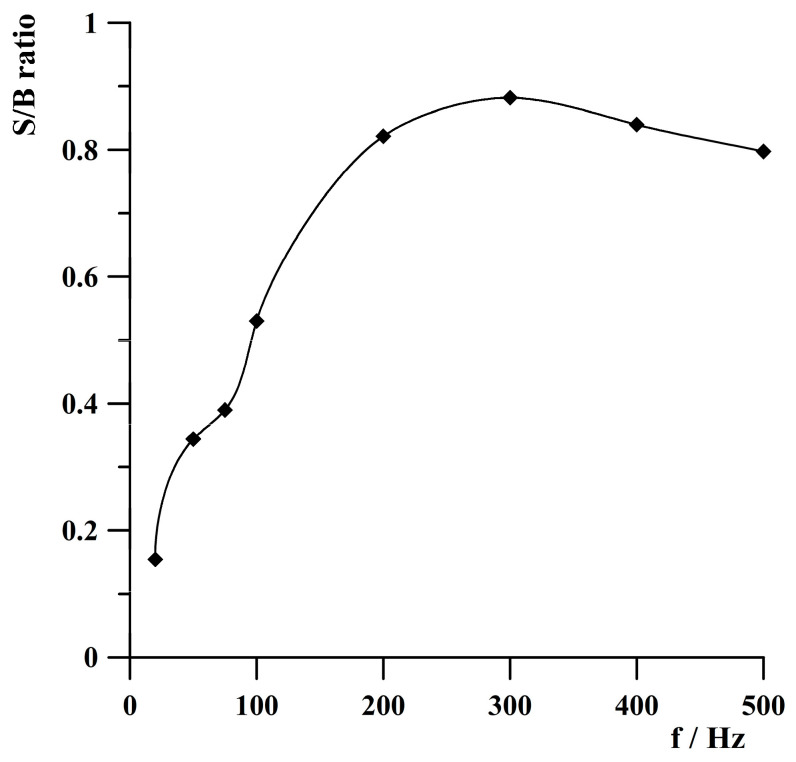
The dependence of the signal-to-background ratio on frequency. Concentration of Tl(I): 2 × 10^−8^ mol·L^−1^. Activation conditions: −3.0 V, 1 s. Deposition conditions: −0.8 V, 120 s. Amplitude and step potential: 25 and 5 mV. S/B—ratio of the thallium signal to the background current value.

The influence of frequency on the thallium square wave voltammetric signal was investigated from 20 to 500 Hz. Thallium(I) concentration was 2 × 10^−8^ mol·L^−1^. Amplitude and step potential were 25 and 5 mV, respectively. It was observed that the thallium peak’s current exhibited almost a linear increase across the entire tested frequency range. Because with increasing frequency, the background current also increases, the optimal frequency value was selected based on the calculated ratio of the thallium signal to the background current value. Based on the obtained results presented in Figure 9, the frequency of 300 Hz was selected for further studies.

### 2.6. Calibration Data

**Figure 10:**
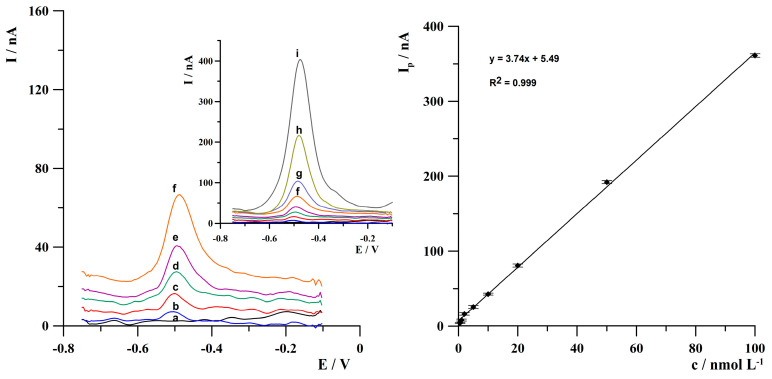
The anodic stripping voltammograms obtained during calibration studies. Concentration of Tl(I): (a) 0; (b) 5 × 10^−10^; (c) 1 × 10^−9^; (d) 2 × 10^−9^; (e) 5 × 10^−9^; (f) 1 × 10^−8^; (g) 2 × 10^−8^; (h) 5 × 10^−8^; (i) 1 × 10^−7^ mol·L^−1^. Activation conditions: −3.0 V, 1 s. Deposition conditions: −0.8 V, 120 s. Frequency, amplitude and step potential: 300 Hz, 25 mV and 5 mV, respectively.

The linear dependence of thallium peak current on its concentration was investigated for 120 s of deposition. The results indicated that the calibration graph was linear from 5 × 10^−10^ to 1 × 10^−7^ mol·L^−1^ of thallium(I) concentration and is expressed by the equation y = 3.74x + 5.49, where y is the peak current (in nA) and x is Tl(I) concentration (in nmol·L^−1^). The correlation coefficient R^2^ was 0.999. The relative standard deviation (RSD) for determining 2 × 10^−8^ mol·L^−1^ of thallium(I) was 3.6% (n = 5). The detection limit (LOD) for Tl(I) determination was calculated as three times the standard deviation of the intercept divided by the slope of the calibration plot. The estimated LOD was 1.35 × 10^−10^ mol·L^−1^. The anodic stripping voltammograms obtained during calibration studies and a calibration graph are shown in Figure 10.

### 2.7. Studies of Repeatability

The repeatability of the peak current obtained for Tl(I) determination at the solid silver microelectrode array was examined. The repeatability expressed as RSD and calculated for the data obtained within one day was calculated based on the thallium peaks’ heights, recorded five times from the same solution containing Tl(I) at a concentration of 2 × 10^−8^ mol L^−1^. The obtained RSD was 3.6%.

Furthermore, it was established that the number of measurements that can be performed from the same solution before the subsequent polishing of the working microelectrode, while maintaining a satisfactory RSD value of 4.5%, is 20. It was therefore concluded that the multiple standard addition method could be employed.

The repeatability examined between days was calculated for the data obtained from different solutions containing 2 × 10^−8^ mol L^−1^ of Tl(I). The obtained RSD was 4.1%. Based on the RSD values obtained, it can be concluded that the repeatability of the procedure is satisfying.

### 2.8. Studies of Interference Effects

The interference effects on thallium peak current were studied in the presence of foreign ions typically present in natural water samples. During these studies, the analyzed solution contained acetate buffer, Na_2_EDTA and thallium(I) at a concentration of 0.05, 2 ×10^−3^ and 2 × 10^−8^ mol·L^−1^, respectively. The obtained results are summarized in Table 2. As can be seen, the determination of Tl(I) was not influenced by most of the tested ions in their 100-fold excess, which proves the high selectivity of the developed analytical procedure. Since in the case of a 100-fold excess of Pb(II) ions and a 50-fold excess of Cu(II) ions, a decrease in the analytical signal of thallium to about 70% of its original value was observed, the use of the standard addition method is recommended. It should be noted that such a high content of foreign ions is rarely found in environmental water samples. The decrease in the thallium peak current in the presence of excess of Pb(II) and Cu(II) is probably connected with a partial covering of deposited thallium by a layer of Pb or Cu, which makes it difficult to oxidize thallium at a potential of its peak.

### 2.9. Analysis of Environmental Water Samples

The proposed procedure with the use of a described silver microelectrode array was successfully applied for Tl(I) determination in certified reference materials TM 25.5 and TM 26.5 (Lake Ontario water sample). Before conducting a proper analysis, the reference materials were diluted (with dilution factors of 5 and 2, respectively) and neutralized with an adequate addition of 2 mol·L^−^^1^ NaOH. The analyses were performed using the standard addition method at deposition time of 120 s (n = 5). The obtained results, presented in Table 3, indicated the possibility of applying the elaborated voltammetric procedure for analyzing natural water samples containing a similar matrix.

The developed procedure of Tl(I) determination was also applied for the analysis of an environmental water sample. Before a proper voltammetric analysis, the water sample was diluted (the dilution factor was 2). The Tl(I) concentration in the water sample collected from Zemborzycki Lake was below the detection limit of the presented procedure, so recovery studies of the sample spiked with known thallium(I) concentrations were carried out. Recovery of Tl(I) at a concentration of 1 × 10^−^^8^ mol·L^−^^1^ was 99.2%, with a relative standard deviation of 3.1%.

**Figure 11:**
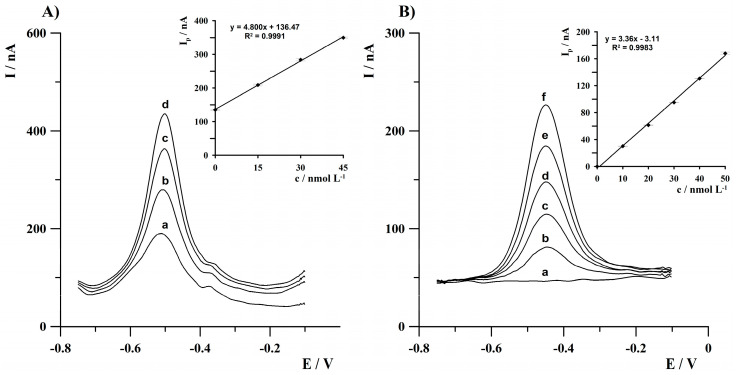
Anodic stripping voltammograms obtained during the analysis of (**A**) certified reference material TM 25.5 and (**B**) Zemborzycki Lake water sample. (**A**) (a) sample of TM 25.5 (dilution factor 5); (b) as (a) + 1.5 × 10^−8^; (c) as (a) + 3 × 10^−8^; (d) as (a) + 4.5 × 10^−8^ mol·L^−1^. (**B**) (a) sample of diluted lake water (dilution factor 2); (b) as (a) + 1 × 10^−8^; (c) as (a) + 2 × 10^−8^; (d) as (a) + 3 × 10^−8^; (e) as (a) + 4 × 10^−8^; (f) as (a) + 5 × 10^−8^ mol·L^−1^. Activation conditions: −3.0 V, 1 s. Deposition conditions: −0.8 V within 120 s. Insets: corresponding standard addition graphs.

The voltammograms obtained during the analysis of certified reference material and natural water samples, as well as the corresponding standard addition graphs, are presented in Figure 11A and Figure 11B, respectively. The above results show that the proposed procedure can be applied for Tl(I) determination in real water samples.

## 3. Materials and Methods

### 3.1. Apparatus

**Figure 12:**
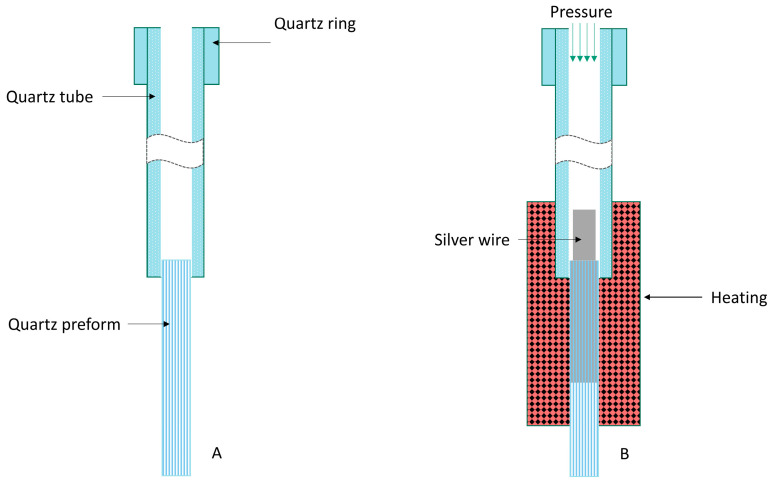
The scheme illustrating the first two steps of fabrication of the silver microelectrode array: (**A**) quartz preform with a spliced quartz tube; (**B**) set for filling a preform with melted metal.

The μAutolab analyzer (Eco Chemie, Utrecht, The Netherlands), controlled by GPES 4.9 software, was used for the voltammetric measurements. A three-electrode electrochemical cell of a volume of 10 mL was used. A solid silver microelectrode array was applied as a working microelectrode. The construction of a working microelectrode array was similar to that described in [33]. Shortly, for the fabrication of an array of silver microelectrodes, a silica preform with 169 holes (purchased from the Faculty of Engineering Tower, University of Malaya, Kuala Lumpur, Malaysia) was used. The outer diameter of the preform was measured to be 3 mm. The holes exhibit a circular shape, with an approximate diameter of 14–16 µm. The minimal distance between the holes was 150 μm. The holes were filled with melted silver in accordance with a procedure similar to those reported in [28,33,34,35,36]. Firstly, at one end of a quartz tube, a quartz ring was spliced to the tube to allow for the closure of the tube and to apply pressure in the course of filling the preform with silver. In the next step, a preform was spliced with the second end of a quartz tube. Then, a silver wire of a diameter of 2 mm was inserted into the quartz tube. Next, the preform mounted to the quartz tube containing silver was closed and inserted into a tube furnace. The part of a preform and the part of a quartz tube were heated to a temperature of about 1080 °C, and the silver wire was melted. Then, the pressure was increased gradually to 10 bars, and the part of the heated preform was filled with melted metal. The first two steps of filling the preform with silver are illustrated in the scheme presented in Figure 12. Next, about 5 mm of a preform filled with silver was polished at both ends and pressed into a PEEK casing. Electrical contact from silver microelectrodes in the array was achieved using graphitized carbon black powder and a copper wire. In this manner, an environmentally sustainable and durable silver microelectrode array, which is suitable for long-term utilization, was obtained.

Before each measurement series, the electrode was polished with sandpaper of 1500 and 2500 grit. After polishing, the silver microelectrode array was cleaned in deionized water using an ultrasonic cleaner (Sonic-3, Polsonic, Warsaw, Poland) for 30 s. As reference and auxiliary electrodes, Ag/AgCl/NaCl_(sat.)_ and a platinum wire were used, respectively.

The real image of the surface of the solid silver microelectrode array was taken using an MA200 Inverted Metallographic Microscope Nikon (Tokyo, Japan).

### 3.2. Reagents

Acetate buffer at a concentration of 1 mol·L^−1^ and a pH of 5.3 was prepared from CH_3_COOH and NaOH Suprapur reagents purchased from Merck. A stock Tl(I) solution at a concentration of 1 g·L^−1^ and foreign ions stock solutions used in interference studies at a concentration of 1 g·L^−1^ were obtained from Fluka (Buchs, Switzerland). The working solutions of Tl(I) at a concentration of 1 × 10^−5^ mol·L^−1^ were prepared from an appropriate dilution of the stock solution in 0.01 mol·L^−1^ HNO_3_. A solution of 0.2 mol·L^−1^ Na_2_EDTA was prepared by dissolution of the reagent purchased from Sigma Aldrich in deionized water. The certified reference materials TM 25.5 and TM 26.5 (Environment and Climate Change, Gatineau, QC, Canada) were used. All chemicals were of analytical reagent grade or Suprapur. All solutions were prepared with deionized water obtained from a Millipore purification system (Millipore, London, UK).

### 3.3. Preparation of the Real Water Sample

A natural water sample from the Zemborzycki Lake (situated in eastern Poland) underwent filtration using a membrane filter (0.45 μm) and was stored in a refrigerator until it was required for further analysis.

### 3.4. Standard Procedure of the Measurements

The analyzed sample was added to the electrochemical vessel. Then, 0.5 mL 1 mol·L^−1^ of acetate buffer (pH 5.3) and 100 µL 0.2 mol·L^−1^ Na_2_EDTA were pipped. Then, deionized water was added to reach a volume of 10 mL. The standard measurement procedure consisted of three main steps:

- The activation step, needed for the working electrode’s surface preparation, was performed by applying a short potential pulse of −3.0 V within 1 s;
- The deposition time step, within which thallium ions underwent reduction to the metallic state on the surface of the microelectrode array, was conducted at a potential of −0.8 V within 120 s;
- After a ten-second equilibration step, a square wave anodic stripping voltammogram was recorded while the potential was changed from −0.8 to −0.1 V. Frequency, amplitude and step potential were 300 Hz, 25 mV and 5 mV, respectively. The research was conducted after a 5-minute solution deoxygenation with nitrogen as an inert gas.

## 4. Conclusions

This article reports for the first time the application of a solid silver microelectrode array for anodic stripping voltammetric determination of Tl(I). Because a non-toxic electrode material was used for its construction, as well as the fact that no surface modification was needed, e.g., with the use of toxic metal ions, the presented sensor is characterized by an eco-friendly character. The method of construction of a solid silver microelectrode array leads to obtaining a sensor with a long service life, which is another benefit in contrast to, e.g., photolithographically fabricated silver microband electrode arrays [37,38], silver nanoparticle-decorated carbon fibre microelectrode [39] or interdigitated microelectrode arrays [40]. The important advantage of the developed procedure is that no surface modification of the microelectrode was used.

The developed procedure is characterized by a wide linear dynamic range from 5 × 10^−10^ to 1 × 10^−7^ mol·L^−1^, a low detection limit of 1.35 × 10^−10^ mol·L^−1^ (for 120 s of deposition), satisfactory selectivity and high repeatability of the obtained results (RSD 3.6%). The results obtained during the analysis of certified reference materials and environmental water sample indicate that the developed procedure can be applied for Tl(I) determination in real water samples containing a similar matrix.

## Figures and Tables

**Table 1:** The comparison of parameters of anodic stripping voltammetric procedures of thallium(I) determination using various working electrodes.

Working Electrode	Deposition Time [s]	Linear Range [nmol L^−1^]	Detection Limit [nmol L^−1^]	Remarks	Ref.
MFE	600	1–500	0.5	-	[13]
BiFE	120	12–150	10.8	rotating disc	[14]
BiFE	240	49–391	2.9	disposability, microfabrication	[15]
BiABE	60	0.5–49	0.005	-	[16]
Bi-graphite electrode	600	49–4900	4.9	mechanically renewed electrode	[17]
BiµE	120	2–200	0.83	solid metal microelectrode	[18]
SbFE	120	9.8–489	4.9	-	[19]
Graphite µE	300	24–1712	0.049	-	[20]
Modified CPE	300	15–1220	4.2	modified with crown ether	[22]
BiF/SPE	60	5–1000	0.847	integrated three-electrode sensor	[24]
BiF/AuµE	120	0.5–500	0.22	-	[28]
GCE/AgNPs-D3	120	49–490	35	modified GCE	[29]
AgµE	120	0.5–100	0.135	unmodified electrode	[this work]

Explanation of abbreviations: MFE—mercury film electrode; BiFE—bismuth film electrode; BiABE—bismuth bulk annular band working electrode; BiµE—bismuth microelectrode; SbFE—antimony film electrode; graphite µE—graphite microelectrode; BiF/SPE—bismuth film screen-printed electrode; BiF/AuµE—solid gold microelectrodes array modified with bismuth film; GCE/AgNPs-D3—glassy carbon electrode modified with silver nanostructures reduced and stabilized with dextrin; AgµE—silver microelectrode array.

**Table 2:** The relative Tl(I) analytical signal in the presence and absence of foreign ions. Tl(I) concentration was 2 × 10^−8^ mol·L^−1^. Deposition conditions: −0.8 V, 120 s.

Foreign Ion	Molar Excess of Foreign Ion	Relative Tl Signal [I/I_0_] ^1^
Ga(III)	100	1.03
Mn(II)	100	0.99
Co(II)	100	1.02
Ni(II)	100	1.04
Cd(II)	100	0.95
Pb(II)	50	0.92
	100	0.72
Cu(II)	20	0.93
	50	0.69
Zn(II)	100	0.94
Fe(III)	100	0.95
In(III)	100	1.04

^1^ Thallium peak current: I—in the presence and I_0_—in the absence of foreign ion.

**Table 3:** Results of Tl(I) determination in certified reference materials TM 25.5 and TM 26.5.

Certified Reference Material	Determined Value ± SD [µg·L^−1^]	Certified Value ± SD [µg·L^−1^]	Recovery [%]
TM 25.5	29.1 ± 1.1	30.0 ± 2.8	97.0
TM 26.5	5.7 ± 0.45	5.41 ± 0.42	105.4

## Data Availability

Data are contained within the article.

## Supplementary Materials

The following supporting information can be downloaded at: https://www.mdpi.com/article/10.3390/molecules30214220/s1, Figure S1: Anodic stripping voltammograms obtained during the study of pH optimization of the procedure of Tl(I) determination. Concentration of Tl(I): 2 × 10^−8^ mol·L^−1^. Activation conditions: −3.0 V, 2 s. Deposition conditions: −0.9 V, 120 s.; Figure S2: The effect of the acetate buffer concentration on the thallium peak current. Concentration of Tl(I): 2 × 10^−8^ mol·L^−1^. Activation conditions: −3.0 V, 2 s. Deposition conditions: −0.9 V, 120 s. The error bars represent the standard deviation (n = 3).; Figure S3: The effect of Na_2_EDTA concentration on the thallium peak current. Concentration of Tl(I): 2 × 10^−8^ mol·L^−1^. Activation conditions: −3.0 V, 2 s. Deposition conditions: −0.9 V, 120 s. The error bars represent the standard deviation (n = 3).; Figure S4: Voltammograms obtained for Tl(I) determination: without the presence of Pb(II) and Na_2_EDTA (black line); in the presence of a 100-fold excess of Pb(II) but without the presence of Na_2_EDTA (blue line); in the presence of a 100-fold excess of Pb(II) and 2 × 10^−3^ mol·L^−1^ Na_2_EDTA (red line). Concentration of Tl(I): 2 × 10^−8^ mol·L^−1^. Activation conditions: −3.0 V, 1 s. Deposition conditions: −0.8 V, 120 s.
