# Supplementary material for: Application of a Novel Solid Silver Microelectrode Array for Anodic Stripping Voltammetric Determination of Thallium(I)
Source: Molecules. 2025 Oct 29;30(21):4220. doi: 10.3390/molecules30214220 (PMC12609488; doi:10.3390/molecules30214220)
Supplement: Supplementary file 1 [file molecules-30-04220-s001.zip › molecules-3916499-supplementary.pdf]

Supplementary Materials

The application of a novel solid silver microelectrode array for anodic stripping voltammetric determination of thallium(I)

Mieczyslaw Korolczuk, Mateusz Ochab, Iwona Gęca*

Institute of Chemical Sciences, Faculty of Chemistry, Maria Curie Sklodowska University, 20-031 Lublin, Poland

* e-mail: iwona.geca@mail.umcs.pl

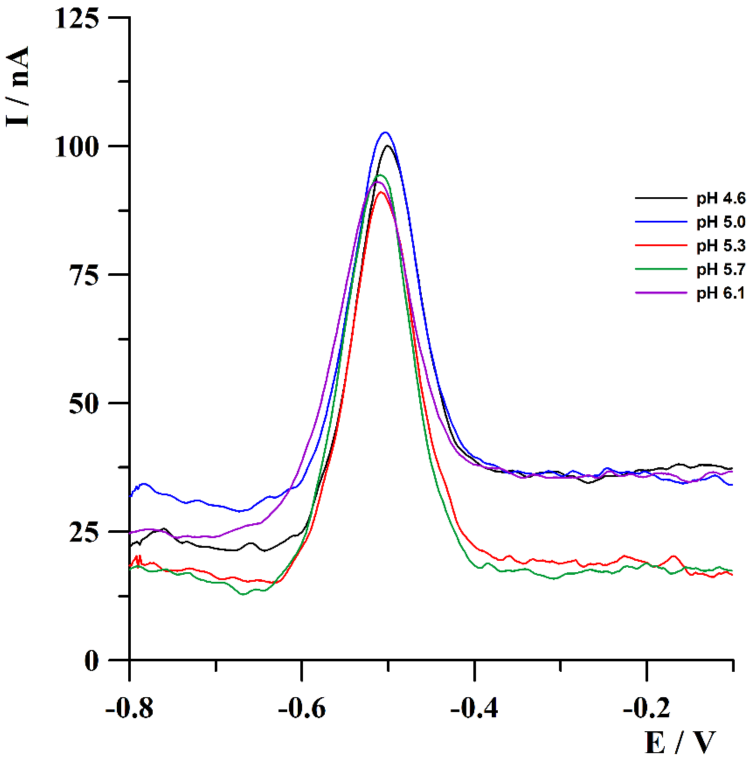

Figure S1: Anodic stripping voltammograms obtained during the study of pH optimization of the procedure of Tl(I) determination. Concentration of Tl(I): 2 × 10^{-8} mol·L^{-1}. Activation conditions: −3.,0 V, 2 s. Deposition conditions: −0.9 V, 120 s.

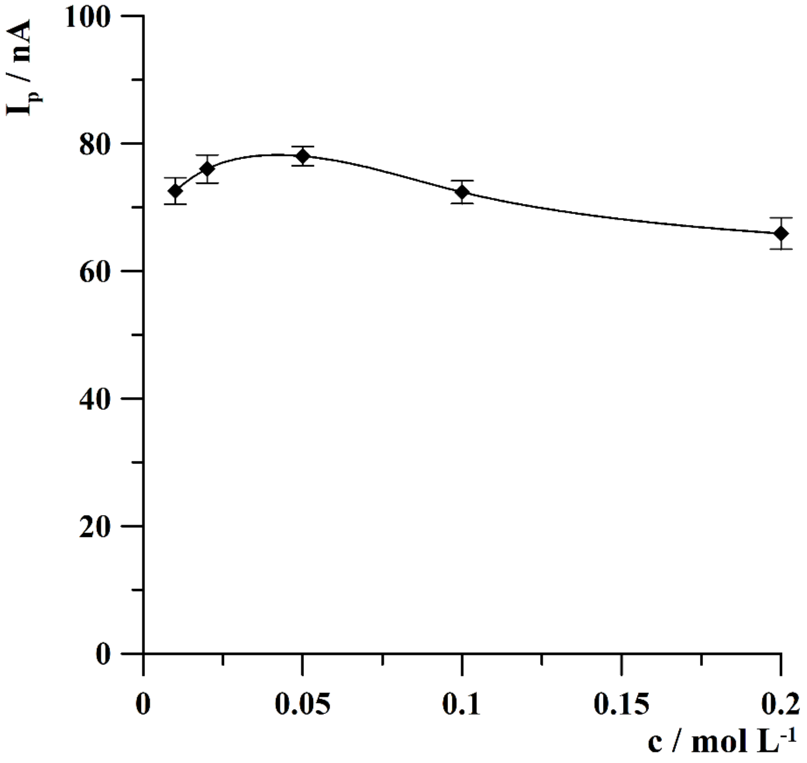

Figure S2: The effect of the acetate buffer concentration on the thallium peak current. Concentration of Tl(I): 2×10^{-8} mol·L^{-1}. Activation conditions: −3.0 V, 2 s. Deposition conditions: −0.9 V, 120 s. The error bars represent the standard deviation (n = 3).

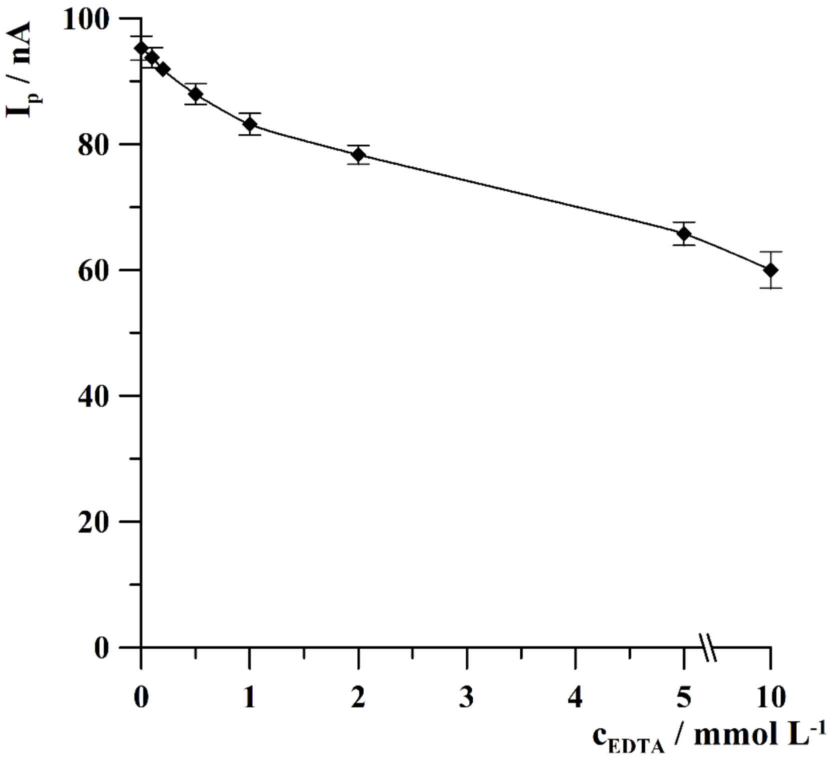

Figure S3: The effect of Na_2EDTA concentration on the thallium peak current. Concentration of Tl(I): 2×10^{-8} mol·L^{-1}. Activation conditions: −3.0 V, 2 s. Deposition conditions: −0.9 V, 120 s. The error bars represent the standard deviation (n = 3).

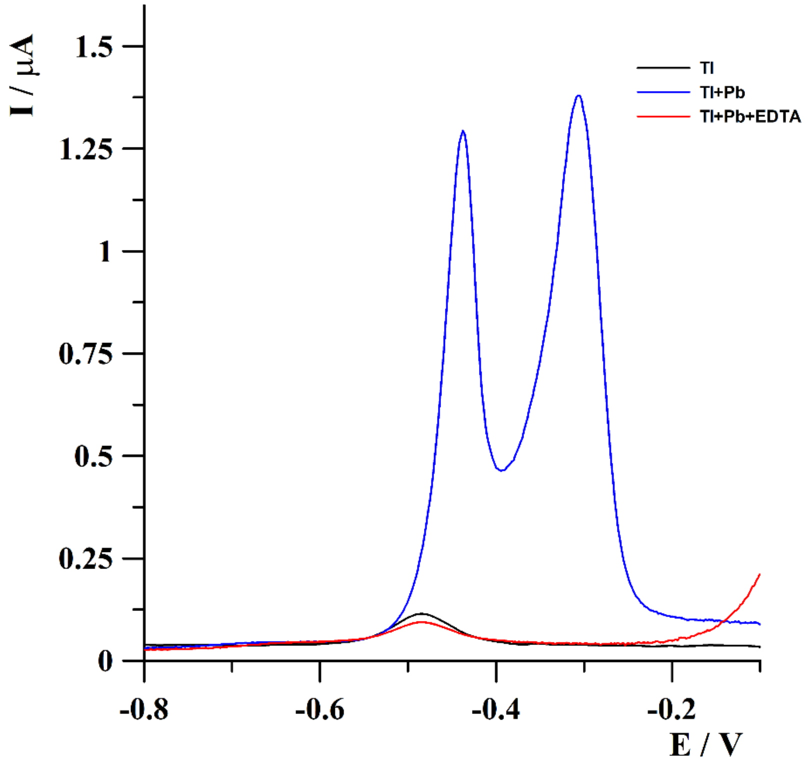

Figure S4: Voltammograms obtained for Tl(I) determination: without the presence of Pb(II) and Na_2EDTA (black line); in the presence of a 100-fold excess of Pb(II) but without the presence of Na_2EDTA (blue line); in the presence of a 100-fold excess of Pb(II) and 2×10^{-3} mol·L^{-1} Na_2EDTA (red line). Concentration of Tl(I): 2×10^{-8} mol·L^{-1}. Activation conditions: −3.0 V, 1 s. Deposition conditions: −0.8 V, 120 s.
